# Supplementary material for: Molecular and Phenotypic Changes in FLExDUX4 Mice
Source: J Pers Med. 2023 Jun 25;13(7):1040. doi: 10.3390/jpm13071040 (PMC10381554; doi:10.3390/jpm13071040)
Supplement: Supplementary file 1 [file jpm-13-01040-s001.zip › jpm-2320277-supplementary.pdf]

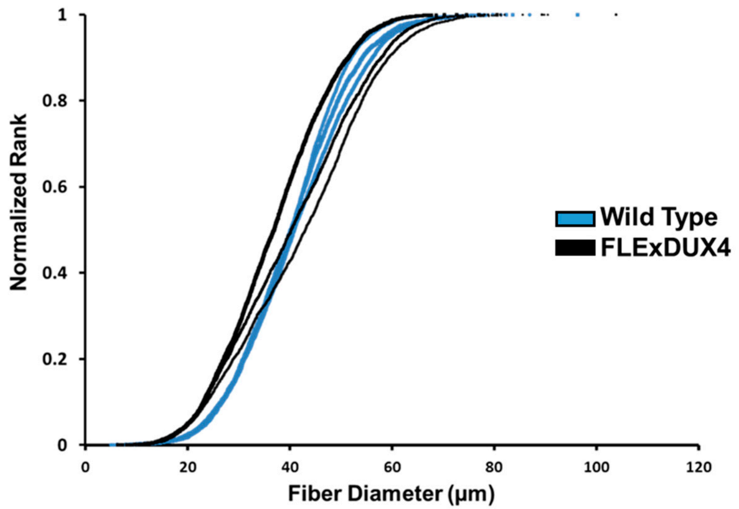

Supplemental Figure S1: Male FLExDUX4 mice display fiber size differences in small fibers at 12 months old of age. Differences in fiber size were determined by comparing Feret's diameters in FLExDUX4 and that in wild-type littermates (n = 3). A total of 3538- 4665 fibers were measured in each section.

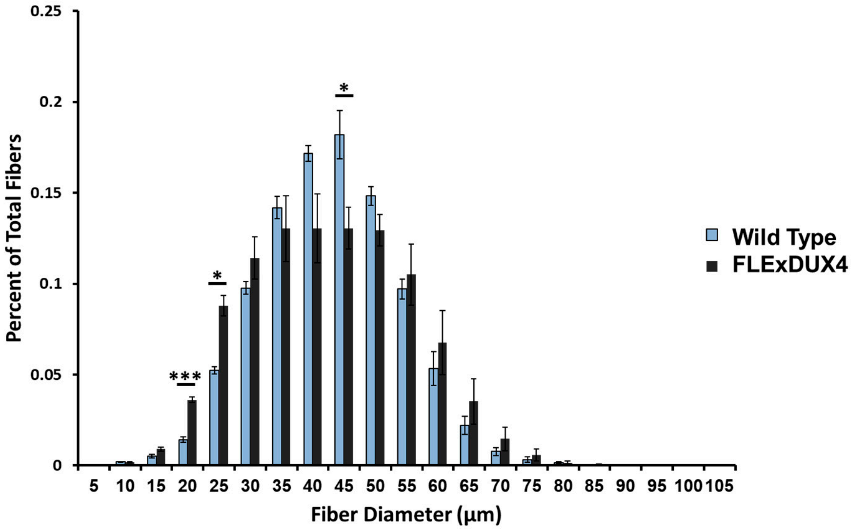

Supplemental Figure S2: Fiber size distribution of the quadriceps muscle in 12-month-old male *FLExDUX4* and wild-type littermates using dystrophin stain. Five fields were taken from a whole section of quadriceps muscle and the muscle fibers were measured in each field. Blue bars denote wild-type littermates. Black bars denote *FLExDUX4* mice. A total of 3538- 4665 fibers were measured for each mouse. Error bars are ± the standard error of the mean. n = 3. *p < 0.05, ***p < 0.001.

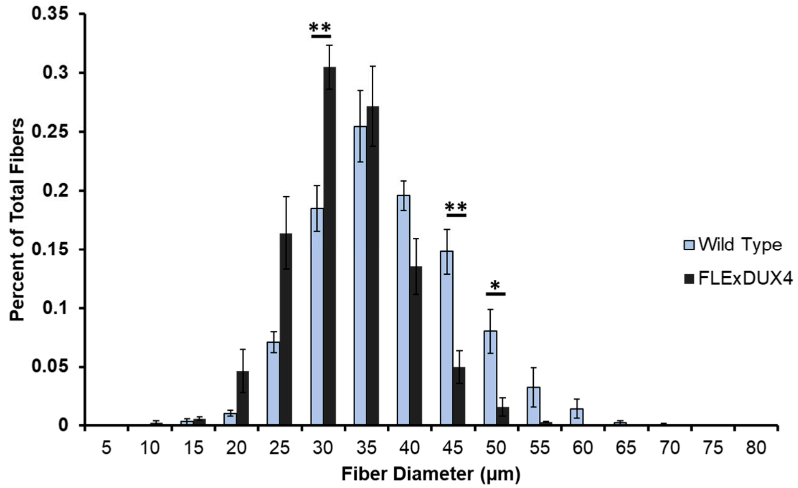

Supplemental Figure S3: Fiber size distribution of the NADH-TR positive fibers in quadriceps in 12-month-old male *FLExDUX4* and wild-type littermates. A total of 586-799 fibers NADH-TR positive fibers were measured for each mouse. Blue bars denote wild type littermates. Black bars denote *FLExDUX4* mice. Error bars are ± the standard error of the mean. n = 3. *p < 0.05, **p < 0.01.

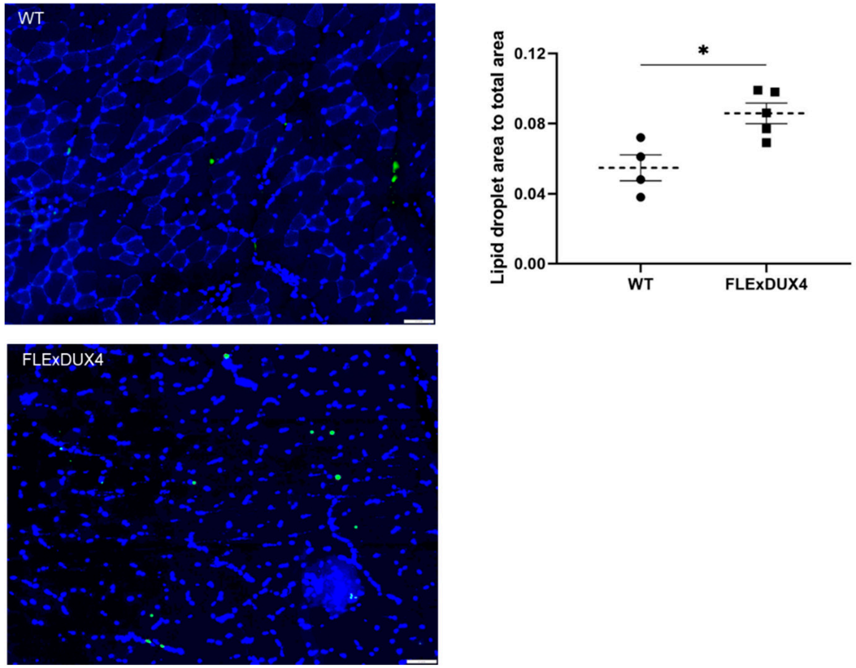

Supplemental Figure S4: BODIPY lipid staining of 4-month-old *FLExDUX4* mice and wild-type littermates. Significantly higher BODIPY staining (green) was observed in the quadriceps *FLExDUX4* mice compared to wild type littermates (wild type n = 4, *FLExDUX4* n = 5). Myonuclei are stained blue. Scale bar measures 50μm.

Supplemental Table S1: Average muscle weights (mg) that were significantly smaller in male *FLExDUX4* mice compared to wild-type littermates at different time points.

	2 months (n=6)			4 months (n=6-7)			8 months (n=5-6)			12 months (n=8-9)		
	**WT**	***FLExDUX4***	**P-value**	**WT**	***FLExDUX4***	**P-value**	**WT**	***FLExDUX4***	**P-value**	**WT**	***FLExDUX4***	**P-value**
**Masseter**	88±3.9	77±3.5	-	110±4.1	104±2.4	-	113±3.0	101±1.6	**	110±5.3	95±5.4	-
**Deltoid**	53±2.6	49±2.6	-	61±2.8	50±3.1	*	63±3.0	55±3.0	-	51±2.8	50±1.2	-
**Triceps**	130±3.9	120±3.1	-	155±4.0	149±2.9	-	153±5.4	140±3.2	-	142±4.9	131±1.7	*
**Biceps**	20±0.5	19±0.5	-	23±0.4	22±0.4	-	24±1.6	21±0.8	-	25±1.0	21±0.7	**
**Gastrocnemius**	149±4.0	138±2.0	*	178±2.5	165±2.9	*	180±5.4	164±4.3	*	182±2.7	157±2.6	***
**Soleus**	9±0.5	8±0.4	*	13±0.7	10±0.4	*	12±1.4	10±0.3	-	14±0.7	10±0.4	***
**Tibialis Anterior**	56±1.2	51±0.9	*	69±1.5	63±1.5	*	60±4.8	64±1.4	-	62±2.2	56±2.0	-
**Quadriceps**	187±7.8	163±4.7	*	230±8.2	200±2.0	*	199±12.8	197±8.3	-	198±7.8	171±5.6	*
**Diaphragm**	93±5.1	88±3.0	-	98±3.4	86±2.9	*	94±5.2	101±3.2	-	103±5.2	91±3.6	-
**Heart**	134±6.5	130±2.0	-	157±4.3	155±6.2	-	153±1.8	162±5.7	-	169±7.5	166±3.5	-

± Standard Error of the Mean *p<0.05, **p<0.01, ***p<0.001.

Supplemental Table S2: Average muscle weights (mg) that were significantly smaller in female *FLExDUX4* mice compared to wild-type littermates at different time points.

	**2 months (n=6)**			**4 months (n=6-8)**			**8 months (n=7-8)**			**12 months (n=6-7)**		
	**WT**	***FLExDUX4***	**P-value**	**WT**	***FLExDUX4***	**P-value**	**WT**	***FLExDUX4***	**P-value**	**WT**	***FLExDUX4***	**P-value**
**Masseter**	58±2.3	57±3.1	-	77±2.1	64±3.0	**	87±1.4	79±2.9	*	89±5.4	76±3.2	-
**Deltoid**	41±1.6	43±7.0	-	45±1.8	38±1.8	*	50±3.1	51±1.3	-	52±2.5	37±1.9	***
**Triceps**	95±2.6	87±3.4	-	107±2.5	98±2.5	*	122±4.5	116±5.0	-	122±5.6	97±5.0	**
**Biceps**	15±0.5	15±0.6	-	19±1.0	18±0.4	-	21±1.0	18±1.2	-	21±1.2	1±0.3	*
**Gastrocnemius**	101±2.9	99±3.0	-	127±2.7	119±3.9	-	140±4.5	139±4.2	-	145±8.0	122±3.6	*
**Soleus**	8±0.5	6±0.4	-	9±0.3	8±0.4	-	10±0.6	9±0.3	-	10±0.4	8.±0.3	*
**Tibialis Anterior**	40±1.0	40±1.1	-	51±1.5	47±1.1	-	55±1.3	55±1.0	-	56±2.0	48±2.2	*
**Quadriceps**	136±3.3	123±6.5	-	173±4.2	151±6.5	*	179±4.9	167±10.6	-	185±12.1	141±4.7	*
**Diaphragm**	71±4.0	74±4.6	-	86±1.9	81±4.4	-	87±6.2	93±4.8	-	112±7.0	95±2.5	-
**Heart**	99±7.2	102±3.0	-	117±3.2	120±3.4	-	119±4.9	135±6.6	-	146±8.7	145±7.8	-

± Standard Error of the Mean, *p<0.05, **p<0.01, ***p<0.001.

Supplemental Table S3: Canonical pathways that are affected in 2-month-old male *FLExDUX4* mice in comparison to wild-type littermates.

Ingenuity Canonical Pathways	P-value	Gene Symbol
**Adipogenesis pathway**	1.3E-4	**Up:** FGFR1,FOXO1, KLF3, LPIN1,LPL,NOCT,PER2<br>**Down:** ARNTL,CLOCK,GTF2H2,KAT6A
**Circadian Rhythm Signaling**	5.9E-4	**Up:** BHLHE40,CRY2,PER2<br>**Down:** ARNTL, CLOCK
**NRF2-mediated Oxidative Stress Response**	2.2E-3	**Up:** BACH1,DNAJB4,FKBP5,FTH1,FTL,GSK3B, MAP2K7<br>**Down:** DNAJB1,FOS, HSPB8,MAF
**Methylmalonyl Pathway**	2.6E-3	**Down:** MCEE,PCCA
**2-oxobutanoate Degradation I**	4.3E-3	**Down:** MCEE,PCCA
**CD27 Signaling in Lymphocytes**	5.0E-3	**Up:** MAP2K7,MAP3K6,NFKBIB,<br>**Down:** FOS, TRAF2
**AMPK Signaling**	5.6E-3	**Up:** ACACB,ADIPOQ,CAMKK2,CCND1,EIF4EBP1,FOXO1,PPM1M, PPP2CA,RAB27A<br>**Down:** IRS1, RPTOR
**Regulation of the Epithelial Mesenchymal Transition by Growth Factors Pathway**	6.6E-3	**Up:** FGF7,FGFR1,FOXO1,GSK3B,IL6R,MAP2K7<br>**Down:** DOCK10,FGF6, FOS, MMP9
**PPARα/RXRα Activation**	7.2E-3	**Up:** ABCA1,ACVR1B,ACVR1C,ADIPOQ,LPL,MAP2K7,NFKBIB<br>**Down:** CLOCK,IRS1,NCOR2,
**April Mediated Signaling**	9.6E-3	**Up:** MAP2K7,NFKBIB,TRAF2<br>**Down:** FOS
